# Development of a self-care program satisfaction questionnaire for cardiovascular patients in Iran

**DOI:** 10.15171/jcvtr.2018.04

**Published:** 2018-03-17

**Authors:** Mehdi Nosratabadi, Zohreh Halvaiepour

**Affiliations:** ^1^Social Determinants of Health Research Center, Isfahan University of Medical Sciences, Isfahan, Iran; ^2^Faculty of Education and Psychology, University of Isfahan, Isfahan, Iran

**Keywords:** Reliability and Validity, Self-care, Cardiac Rehabilitation, Scale Development

## Abstract

***Introduction:*** Evaluating patient satisfaction with self-care program can increase the likelihood of complying with treatment. There is no valid and reliable tool to be used in measuring Iranian patient’s satisfaction with self-care program. So, this study intends to develop the patient satisfaction questionnaire in the context of cardiac rehabilitation and test its validity and reliability in Iranian patients.

***Methods:*** A cross-sectional study was conducted to develop and validate the patient satisfaction with self-care program questionnaire using structural modeling. A total of 155 cardiovascular patients referring to cardiovascular rehabilitation center in Isfahan were participated in this study. Construct and criterion validity, and test-retest reliability were used to validate the scale.

***
Results:*** After reviewing literature and receiving expert’ comments for items pooling as well as conducting exploratory factor analysis, 10 statements in the model remained which are loaded on 2 factors. These 2 subscales explained about 63 percent of variance of all constructs. The Cronbach’s alpha coefficient ranged between 0.87 and 0.89 for the whole questionnaires and its subscales. Besides, scale had excellent stability (intraclass correlation = 0.86). Criterion validity analyzed through correlational analyses revealed significant relationships between the current scale and Patient Satisfaction Questionnaire Short Form (PSQ11). CFA revealed an acceptable overall fit for two-factor model.

***Conclusion:*** The scale integrated 10 items in two dimension including patient satisfaction with rehabilitation program and patient satisfaction with personnel of rehabilitation team. In total, most of the psychometric properties of the 10-item patient satisfaction with self-care program scale achieved the standard level and were sufficient to recommend for cardiac rehabilitation settings.

## Introduction

Cardiovascular diseases are the most widespread cause of death in Iran.^1^ It is estimated that cardiovascular disease in Iran accounts for about 46% of all major causes of death.^2^ Studies have shown that the disability-adjusted life year related to Cardiovascular diseases in Iran will increase more than two-fold in 2025 compared with 2005.^3^

It seems compliance with treatment can be mostly resulted from patient’ satisfaction of self-care program. In fact, lack of satisfaction has known as one barriers to practice self-care program.^4^ Moreover, the role of personnel in rehabilitation team should be considered. Patient satisfaction with personnel and program content is accompanied with important outcomes, such as improved quality of health care services^5^ superior compliance and better prognosis.^6^

Several tools have been developed to measure satisfaction with self-care program around the world, including Cardiac Rehabilitation Preference Form^7^ as well as Seattle Angina Questionnaire.^8^ In Iran, some studies have been conducted regarding self-care models in cardiovascular disease^9,10^ but, there is no tool for measuring satisfaction with self-care program in Iran. Understanding and determining the concept of satisfaction based on social differences is essential for promoting positive health outcomes in Iranian cardiovascular patients. Furthermore, no study has yet addressed developing the tool of Patient satisfaction with self-care program in Iranian context, particularly in cardiac rehabilitation centers. So there is an important gap in this regard. Due to context of Iran, it is essential to develop a psychometrically valid and reliable questionnaire to measure satisfaction with self-care programs in the field of cardiac rehabilitation.

## Materials and Methods

This study is a cross-sectional design using structural model aimed to construct and validate satisfaction with self-care program scale.

### Participants and Procedure

The study population consisted of patients who were referring to cardiovascular research center in Isfahan, in this study 155 patients with coronary heart disease, who were referring to cardiovascular research center in Isfahan(Chamran Cardiology hospital), were randomly recruited (from October 2016 to March 2017)

Participants were eligible if they were attended in self-care education program during three month ago and willing to participate in study.

The inclusion criteria include having attended in self-care education program, aged 18 years or above and having the ability and willingness of patients to participate

### Face and content validity

After reviewing the different literature regarding satisfaction with self-care program in cardiac rehabilitation setting (in the sources such as PubMed and Science Direct, with Keywords including cardiac rehabilitation, self-care program satisfaction 25 initial items were extracted. These statements adopted in a culturally acceptable manner based on experts’ points of view. Experts involved in this study had experienced in the cardiac rehabilitation-related fields namely cardiovascular specialist, Nutritionist, psychologist, social worker and nurse. Consequently, 10 items omitted and the final items pool contained 15 items.

The content validity of the final questionnaire was determined by calculating CVI index according to the clarity, relevancy, simplicity, and consistency of each question with the questions set from 10 experts in the field of cardiac rehabilitation. Changes were made to the tools and content validity of the questionnaires was finally confirmed. Therefore, the final questionnaire consisted of 10 questions.

### Criterion validity: Concurrent validity

To determine concurrent validity, the correlation was assessed between current questionnaire and The Patient Satisfaction Questionnaire Short Form (PSQ11).^11^ This 11-items Likert scale questionnaire recommended seven dimensions of patient satisfaction directed toward their doctors and was developed through rigorous research and abbreviated from much larger questionnaires maintaining internal consistency and reliability

### Construct validity

An exploratory factor analysis (principal components analysis followed by a Varimax rotation) was used to prove the construct validity.

It was assumed that the model can be considered satisfactory when the χ²/*df* is lower than 2, RMSEA and SRMR lower than 0.08, CFI, GFI and AGFI higher than 0.90.^12^

### Reliability: internal consistency and repeatability

In our study internal consistency was assessed by Cronbach’s alpha (should be >0.07) for 150 patients. We employed the test-retest method to evaluate the repeatability of the questionnaire. To do so, 25 patients completed this scales twice (at 2-week intervals). To interpret the achieved coefficients values above 0.7 were considered as excellent reliability.^13^ Data were analyzed using SPSS Software. And structural equation analysis with latent variables (SEM) using AMOS module 20.0 was employed.

## Results

### Descriptive statistics

In this study, out of all participants, 112 (72.3%) were male and 43 (27.7%) were female. The mean age of them was 57.9 ± 9.40. 153 (98.7%) were married. 132(83.0%) were diploma and lower than diploma.

### Factorial validity

The KMO and Bartlett’s test equaled 0.85 and 0.001 respectively. As mentioned above, the final version of the questionnaire consisted of 10 statements which be placed in two dimensions including patient satisfaction with rehabilitation program (6 items) and patient satisfaction with personnel of rehabilitation team( 4 items) according to exploratory factor analysis. These two subscales explained 68.6% of the variance in the data that can be considered reasonable for a questionnaire (Table 1).

**Table 1:** Factor analysis of patient satisfaction questionnaire with self-care program

**Number**	**Item**	**Loading Factor**
**Patient satisfaction with rehabilitation program (6 items, eigenvalue = 4, accounts for 38.6 of variance)**		
P.P1	Actions related to the self- care program is easy and I can do it well.	0.84
P.P2	In general self-care program is a useful program to recover from the disease.	0.84
P.P3	I am able to fully implement self-care program and continuing care recommendations.	0.79
P.P4	Self-care program has met the supportive and facilitative role of family in the continuity of self-care	0.79
P.P5	Self-care program is cost-effective and affordable. So I can afford to do it.	0.83
P.P6	as part of the process of treatment, I am aware of self-care program and have accepted it correctly	0.63
**Patient satisfaction with personnel of rehabilitation team (4 items, eigenvalue=4, accounts for 29.9 of variance)**		
P.T1	Description and training of health care team about what I need to know regarding self-care, such as physical activities, stress management and sleep, had been sufficient.	0.82
P.T2	I have been informed by self-care personnel team about psychological and Career counseling needed for better effectiveness of program.	0.85
P.T3	I am satisfied with follow-up made by personnel of self-care program about my disease complications and concerns.	0.82
P.T4	I have been informed and compelled by self-care personnel team about ‏.the reason of conducting self-care program and provided equipments.	0.86

**Figure 1:**
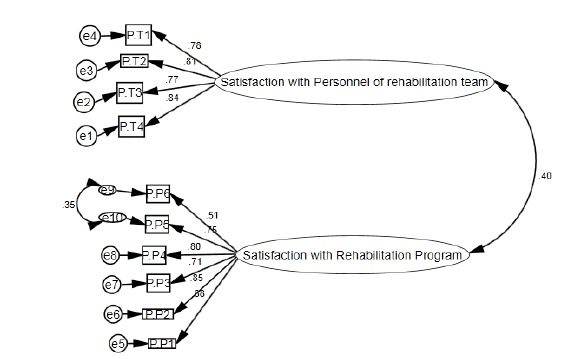
Confirmatory factor analysis of Patient Satisfaction questionnaire with self-care program.
Note. Model fit indices: χ²/df = 1.9; RMSEA = 0.07(90% CI: 0.05 -0.1) *P* close to fit <0.05; GFI = 0.99, AGFI = 0.92 and CFI = 0.96. All CFA loadings estimates were standardized and significant at *P*<0.05.

In addition to exploratory factor analysis, confirmatory factor analysis was made on data. This analysis revealed that the two-factor model provided a good fit to the data (χ²/df = 1.9, *P* < 0.05; RMSEA = 0.07 (90% CI: 0.05 - 0.1), *P* close to fit <0.05; GFI = 0.99, AGFI = 0.92 and CFI = 0.96) (Figure 1).

### Reliability and concurrent criterion validity

Examination of Cronbach’s alpha highlighted good internal consistency for the whole scale (α = 0.87) and for each factor: “Patient satisfaction with rehabilitation program” (α = 0.89) and ”Patient satisfaction with personnel of rehabilitation” (α = 0.87). Moreover, conducting test-retest method to evaluate the repeatability of the questionnaire revealed good results (Table 2).

**Table 2:** Internal consistency and Intra-class correlation (ICC) of the subscales and total scale

**Domain**	**ICC (95% CI) (n=25)**	**Cronbach's alpha** ^*^ ** (n=155)**
Patient satisfaction with rehabilitation program	0.87 (0.86-0.91)	0.89
Patient satisfaction with personnel of rehabilitation	0.85 (0.84-0.90)	0.87
Total	0.86 (0.83-0.90)	0.87

Concurrent criterion validity was computed by correlating the total and subscale score of questionnaire in current study with scores of Patient Satisfaction Questionnaire Short Form (PSQ11). According to the results, patient satisfaction (total score), patient satisfaction with rehabilitation program and patient satisfaction with personnel of rehabilitation team were significantly positive and moderate correlation with PSQ (ranged 0.57, 0.40 and 0.61 respectively).

## Discussion

As the results showed, after initial reviewing and content analysis, the scale integrated 10 items in two dimension which can account for 68.5% of variance in total questionnaire.

Confirmatory factor analysis of questionnaire revealed satisfactory fitness. Regarding reliability, the result shows good internal consistency in two subscales and total score. Besides, test-retest correlation coefficient was reported satisfactory indicating good repeatability.

In developing tools such as satisfaction with the cardiac rehabilitation program, some studies have used cross-sectional study as current study^14^ in other studies prospective cohort research design^15^ or interventional design^16^ were employed. In all of these studies, satisfaction with rehabilitation program and satisfaction with personnel of rehabilitation team are among the important factors of cardiac rehabilitation program.

Outpatient satisfaction in rehabilitation scale^17^ compared with our questionnaire, does not measure satisfaction with Personnel of rehabilitation team, but newer version of this scale^18^ measure a broader range of aspects, including satisfaction with care program, with good psychometric properties.

Seattle Angina Questionnaire, as comparable questionnaire with developed one in current study, does not measure some dimensions such as other aspects of satisfaction with personnel of rehabilitation team. Studies also showed no significant difference in patient satisfaction attending in cardiac rehabilitation program.^15,19^ So it is not clear that this questionnaire is enough much effective in cardiac rehabilitation settings.

Compared with other questionnaires regarding cardiac rehabilitation, we can refer to Cardiac Rehabilitation Preference Form.^7^ This instrument was developed to measure the extent to which client preferences for specific CR program features were met in both the importance and the experience measure, so this scale, in this regard differs from our questionnaire.

## Conclusion

Overall, by developing one 10-item tool comprising two subscales, this study showed that questionnaire of patient satisfaction with self-care program has satisfactory psychometric properties in sample of Iranian patients referring to cardiac rehabilitation center.

## Competing interests

Authors declare no conflict of interest in this study.

## Ethical approval

This study was approved by Ethics Committee of the Isfahan University of Medical Sciences.
